# Left Ventricular Systolic Dysfunction Is a Possible Independent Risk Factor of Radiation Pneumonitis in Locally Advanced Lung Cancer Patients

**DOI:** 10.3389/fonc.2019.01511

**Published:** 2020-01-24

**Authors:** Guoxin Cai, Shuai Liang, Chuanbao Li, Xue Meng, Jinming Yu

**Affiliations:** ^1^Department of Radiation Oncology, School of Medicine, Shandong University, Jinan, China; ^2^Department of Radiation Oncology, Shandong Cancer Hospital Affiliated to Shandong First Medical University, Shandong Academy of Medical Science, Jinan, China; ^3^Department of Emergency, Chest Pain Center, Qilu Hospital of Shandong University, Jinan, China

**Keywords:** left ventricular ejection fraction, radiation pneumonitis, left ventricular systolic dysfunction, locally advanced lung cancer, definitive radiotherapy

## Abstract

**Objectives:** To assess the association between left ventricular (LV) systolic and diastolic dysfunction and grade ≥2 radiation pneumonitis (RP) for locally advanced lung cancer patients receiving definitive radiotherapy.

**Materials and Methods:** A retrospective analysis was carried out for 260 lung cancer patients treated with definitive radiotherapy between 2015 and 2017. RP was evaluated according to Radiation Therapy Oncology Group (RTOG) toxicity criteria. Logistic regression analysis, 10-fold cross validation, and external validation were performed. The prediction model's discriminative performance was evaluated using the area under the receiver operating characteristic curve (AUC), and calibration of the model was assessed by the Hosmer-Lemeshow test and the calibration curve.

**Results:** Within the first 6 months after radiotherapy, 70 patients (26.9%) developed grade ≥2 RP. Reduced left ventricular ejection fraction (LVEF) before radiotherapy was detected in 53 patients (20.4%). The odds ratio (OR) of developing RP for patients with LVEF <50% was 3.42 [*p* < 0.001, 95% confidence interval (CI), 1.85–6.32]. Multivariate analysis showed that forced expiratory volume in the first second/forced vital capacity (FEV1/FVC), LVEF, Eastern Cooperative Oncology Group (ECOG) performance status, chemotherapy, and mean lung dose (MLD) were significantly associated with grade ≥2 RP. The AUC of a model including the above five variables was 0.835 (95% CI, 0.778–0.891) on 10-fold cross validation and 0.742 (95% CI, 0.633–0.851) on the external validation set. The *p*-value for the Hosmer-Lemeshow test was 0.656 on 10-fold cross validation and 0.534 on the external validation set.

**Conclusion:** LV systolic dysfunction is a possible independent risk factor for RP in locally advanced lung cancer patients receiving definitive radiotherapy.

## Introduction

Concurrent chemoradiotherapy is a standard treatment regimen for patients with inoperable locally advanced non-small cell lung cancer (NSCLC) (1) or small cell lung cancer (2). Radiation pneumonitis (RP) is an important dose-limiting complication of thoracic radiotherapy, with clinical symptoms of dyspnea, a non-productive cough, and occasionally low-grade transient fevers (3). Risk factors for RP are various, including lung volume receiving ≥20 Gy, mean lung dose (MLD), chemotherapy, age, former or current smoker, chronic obstructive pulmonary disease, and interstitial lung disease (4–9).

Previous preclinical (10–12) and clinical (13) studies have demonstrated that heart irradiation increases the risk of radiation-induced pulmonary dysfunction. The possible mechanism is that heart radiation directly leads to perivascular fibrosis and myocardial damage and therefore increases end-diastolic pressure, contributing to left ventricular (LV) diastolic dysfunction, which further leads to pulmonary interstitial edema (12), suggesting a detrimental effect of reduced ventricular function on lung tissue. Nalbantov et al. (14) demonstrated cardiac comorbidity was an independent risk factor for developing radiation-induced lung toxicity in lung cancer patients receiving definitive radiotherapy. However, cardiac comorbidity is only a qualitative indicator that couldn't quantify detailed LV systolic and diastolic function. Semrau et al. (15) found that for patients with inoperable NSCLC receiving concurrent chemoradiotherapy, left ventricular ejection fraction (LVEF) ≤50% had no significant association with grades III and IV RP according to the Common Toxicity Criteria. But the overall incidence of RP was fairly low: only three out of 130 patients (2.3%), therefore it couldn't accurately reflect the association between baseline cardiac function and RP.

In the present study, we aimed to evaluate the relationship between LV systolic and diastolic dysfunction and grade ≥2 RP.

## Materials and Methods

### Patients

A retrospective analysis was carried out for patients with stages IIA–IIIB lung cancer and Eastern Cooperative Oncology Group (ECOG) performance status of zero to two treated with definitive radiotherapy at Qilu Hospital of Shandong University between January 2015 and December 2017. Those who received palliative, preoperative or post-operative radiotherapy, could not complete radiotherapy regimen, or had secondary primary tumor were excluded from this study. Tumor was staged according to the American Joint Committee of Cancer seven edition criteria. Before treatment, contrast-enhanced thoracic and abdominal computed tomography, whole-body bone scan, and brain MRI were performed for all patients. Fluorodeoxyglucose positron emission tomography/computed tomography was optional for enrolled patients. Biopsy methods included bronchoscopy, thoracoscopy, or percutaneous transthoracic needle biopsy. Patients in the external validation set were collected from Shandong Provincial Hospital between 2015 and 2017. This study was approved by the Ethics Committee of Qilu Hospital of Shandong University and Shandong Provincial Hospital, and informed consents were obtained from all included individuals.

### Treatment

Treatment-planning computed tomography scans using intravenous contrast were performed for all patients. The radiotherapy was delivered at 2 Gy per fraction daily and five fractions per week by using linear accelerators with an energy of 6-MV or 10-MV X-ray. The prescribed dose ranged from 60 to 70 Gy. Radiation techniques included three-dimensional conformal radiation therapy or intensity-modulated radiation therapy. We set normal tissue tolerance dose-limits according to the National Comprehensive Cancer Network guidelines (16): maximum dose to spinal cord ≤50 Gy, lung volume receiving ≥20 Gy ≤35%, lung volume received ≥5 Gy ≤65%, MLD ≤20 Gy, heart volume received ≥40 Gy ≤80%, mean heart dose (MHD) ≤35 Gy, and esophagus mean dose ≤34 Gy. Most patients received concurrent or sequential chemotherapy. For patients with NSCLC, the regimens consisted of two cycles of platinum-based chemotherapy. For patients with small cell lung cancer, the regimens were two cycles of etoposide plus platinum. Detailed information about treatment plan was collected from medical records.

### Evaluation of Cardiac Function

All patients underwent a comprehensive transthoracic echocardiographic evaluation using GE Vivid 7 (GE Healthcare, Horten, Norway) and pulmonary function testing within 1 week before receiving radiotherapy. Consistent with the American Society of Echocardiography guidelines (17), echocardiography including conventional pulsed-wave Doppler and tissue Doppler echocardiography was performed to acquire transvalvular flow and tissue Doppler recordings. LVEF was used to assess LV systolic function. A cut-off of 50% was regarded as the normal limits for LVEF. For assessing diastolic function, an algorithm recently put forward by Mitter et al. (18) was used. This algorithm uses left atrial volume index (LAVI) >28 ml/m^2^ and age-related reduced lateral early diastolic mitral annular velocity (e′) to check for presence of diastolic dysfunction. The key variables for grading of LV diastolic dysfunction include the ratio of early diastolic transmitral flow velocity to late diastolic transmitral flow velocity (E/A), e′, LAVI, and E-wave deceleration time. The details for grading of LV diastolic function are as follows: normal: E/A > 0.8, e′ normal for age, normal LAVI; grade I: E/A < 0.8, reduced e′ for age; grade II: E/A > 0.8, reduced e′ for age, LAVI > 28 ml/m^2^; grade III: E/A > 1.5, reduced e′ for age, E-wave deceleration time <140 ms, LAVI >28 ml/m^2^. Pulmonary and cardiac comorbidities were defined as a recorded historical treatment of any pulmonary and cardiac disorders at hospital before the start of radiotherapy, irrespective of their severity. Given that the assessment method for LV diastolic function by Mitter et al. (18) was not applicable to tachycardia, bradycardia, atrioventricular block, arrhythmia, mitral annular calcification, mitral valve prosthesis, any mitral stenosis, and ≥3+ mitral regurgitation, patients with these cardiac comorbidities were excluded from our study. Before treatment, baseline dyspnea score [Common Terminology Criteria for Adverse Events version 3.0 (19)] was assessed for all patients and New York Heart Association classification was reassessed for patients with cardiac comorbidity, with baseline dyspnea score >2 and New York Heart Association grade >3 being excluded. A cut-off of 70% was regarded as the normal limits for forced expiratory volume in the first second/forced vital capacity (FEV1/FVC).

### Evaluation of Pulmonary Toxicity and Follow-Up

The primary endpoint was grade ≥2 RP. Early RP usually occurs 1–6 months after radiotherapy, whereas late lung fibrosis usually occurs 6–24 months after radiotherapy (9, 20, 21). Because we focused on early RP, we used 6 months as the cut-off for diagnosis. RP was graded according to the Common Terminology Criteria for Adverse Events v3.0. A diagnosis of RP was based on clinical symptoms and/or radiograghic changes and was determined with consensus by at least two radiation oncologists. Radiograghic changes include ground-glass opacities and/or consolidation and nodular and focal consolidative opacities within the treatment filed. Radiographic changes in RP outside the treatment portals may also appear (22). Clinical symptoms include dyspnea, dry cough, and low-grade fever (4). Cases difficult to diagnose were referred to a respiratory physician or cardiologist to exclude other diseases. Patients were evaluated by radiation oncologists fortnightly during radiotherapy and once a month thereafter until 6 months after radiotherapy. CT scans were performed at each follow-up visit.

### Statistical Analysis

Univariate and multivariate analyses were performed on the training set and validated on the validation set. Variables which were statistically significant in univariate analysis were included in multivariate analysis. The association between variables and RP in univariate analyses was assessed using a chi-square test. Variables significantly associated with grade ≥2 RP in multivariate analysis were put into a model to predict grade ≥2 RP. Internal validation was performed with 10-fold cross-validation for the training set. External validation was performed with data from Shandong Provincial Hospital. The model's discriminative ability was evaluated using the area under the receiver operating characteristic curve (AUC), and calibration of the model was assessed by the Hosmer-Lemeshow test and the calibration curve (23). *P*-values for the difference between AUC for the 10-fold cross validation or the external validation and AUC = 0.05 (random model) were calculated using 1,000 bootstrap samples. For all analyses, *p* < 0.05 were considered statistically significant. The univariate and multivariate logistic regression analyses were performed by SPSS Statistics Version 23.0 (IBM Corporation, Armonk, NY, USA), and the evaluation methods of the model performance were performed using R (version 3.6.1).

## Results

Two hundred and sixty patients and one hundred and twenty patients were enrolled in the training and validation set, respectively. Seventy patients (26.9%) on the training set and 33 patients (27.5%) on the validation set developed grade ≥2 RP within 6 months after radiotherapy. Patient characteristics on the training and validation set are listed in Table 1.

**Table 1:** Patient characteristics.

**Characteristic**	**Patients (%)**	
	**Training set**	**Validation set**
Gender		
Male	172 (66.1)	88 (73.3)
Female	88 (33.9)	32 (26.7)
Current or former smoker		
Yes	144 (55.4)	62 (51.7)
No	116 (44.6)	58 (48.3)
Pulmonary comorbidity		
Yes	58 (22.3)	27 (22.5)
No	202 (77.7)	93 (77.5)
Cardiac comorbidity		
Yes	53 (20.4)	22 (18.3)
No	207 (79.6)	98 (81.7)
^*^NYHA classification		
Grade 1	13 (24.5)	7 (31.8)
Grade 2	23 (43.4)	9 (40.9)
Grade 3	17 (32.1)	6 (27.3)
ECOG Performance status		
0	95 (36.5)	50 (41.7)
1	148 (56.9)	61 (50.8)
2	17 (6.6)	9 (7.5)
Baseline dyspnea score		
0	108 (41.5)	54 (45.0)
1	125 (48.1)	53 (44.2)
2	27 (10.4)	13 (10.8)
Histology		
Squamous cell carcinoma	98 (37.7)	45 (37.5)
Adenocarcinoma	110 (42.3)	45 (37.5)
Small cell carcinoma	52 (20.0)	30 (25.0)
Tumor location		
Upper lobe	142 (54.6)	72 (60.0)
Lower/middle lobe	118 (45.4)	48 (40.0)
cT-stage		
T2	59 (22.7)	32 (26.7)
T3	181 (72.0)	82 (68.3)
T4	20 (5.3)	6 (5.00)
cN-stage		
N0	21 (8.1)	13 (10.8)
N1	126 (48.5)	51 (42.5)
N2	96 (36.9)	43 (35.8)
N3	17 (6.5)	13 (10.9)
TNM stage		
IIA	25 (9.6)	10 (8.3)
IIB	35 (13.5)	19 (15.8)
IIIA	171 (65.8)	78 (65.0)
IIIB	29 (11.1)	13 (10.8)
Treatment modality		
Concurrent chemotherapy	131 (50.4)	64 (53.3)
Sequential chemotherapy	73 (28.1)	18 (15.0)
No chemotherapy	56 (21.5)	38 (31.7)
Chemotherapy regimens		
NSCLC	208 (80.0)	90 (75.0)
cisplatin/carboplatin + etoposide	66 (25.4)	26 (21.7)
cisplatin/carboplatin + paclitaxel	58 (22.3)	20 (16.7)
cisplatin/carboplatin + pemetrexed	50 (19.2)	21 (17.5)
cisplatin/carboplatin + vinorelbine	20 (7.7)	11 (9.1)
cisplatin/carboplatin + gemcitabine	14 (5.4)	12 (10.0)
SCLC	52 (20.0)	30 (25.0)
cisplatin/carboplatin + etoposide	52 (20.0)	30 (25.0)
Radiotherapy technique		
3D-CRT	104 (40.0)	51 (42.5)
IMRT	156 (60.0)	69 (57.5)
	Median (SD)	
Age at lung cancer diagnosis, year	64 (8.48)	63 (8.92)
Prescribed radiation dose, Gy	64 (2.53)	64 (3.25)
Mean lung dose, Gy	16.65 (6.01)	15.75 (5.81)
FEV1/FVC, %	75.80 (12.04)	77.65 (13.43)
FS, %	33.62 (5.17)	34.68 (5.26)
SV, ml	56.83 (11.09)	59.49 (10.41)
LVEF, %	60 (8.22)	55 (6.28)
E/A	1.03 (0.19)	1.10 (0.18)
E/e′	6.09 (3.42)	6.23 (3.84)

*NYHA, New York Heart Association; ECOG, Eastern Cooperative Oncology Group; 3D-CRT, three-dimensional conformal radiation therapy; IMRT, intensity-modulated radiation therapy; SD, standard deviation; FEV1/FVC, forced expiratory volume in the first second/forced vital capacity; FS, fractional shortening; SV, stroke volume; LVEF, left ventricular ejection fraction; E/A, the ratio of early diastolic transmitral flow velocity to late diastolic transmitral flow velocity; E/e′, the ratio of early diastolic transmitral flow velocity to early diastolic mitral annular velocity*.

* *The NYHA classification was assessed only for patients with cardiac comorbidity*.

In univariate analysis, pulmonary comorbidity, FEV1/FVC, cardiac comorbidity, LVEF, LV diastolic dysfunction, ECOG performance status, concurrent or sequential chemotherapy, and MLD were significantly associated with grade ≥2 RP (Table 2). FEV1/FVC [hazard ratio (HR), 6.02; 95% confidence interval (CI), 2.13–17.00; *p* = 0.001], LVEF (HR, 3.38; 95% CI, 1.43–7.96; *p* = 0.005), ECOG performance status (HR, 4.38; 95% CI, 1.76–10.89; *p* = 0.001), concurrent or sequential chemotherapy (HR, 3.08; 95% CI, 1.40–6.78; *p* = 0.005), and MLD (HR, 5.08; 95% CI, 2.39–10.82; *p* < 0.001) remained significant difference in multivariate analysis. The odds ratio of developing RP for patients with LVEF <50 was 3.42 (95% CI, 1.85–6.32; *p* < 0.001) on the training set and 3.00 (95% CI, 1.13–7.97; *p* = 0.023) on the validation set (Table 3).

**Table 2:** Univariate and multivariate logistic regressions analysis for grade ≥2 radiation pneumonitis.

**Characteristic**	**Univariable analysis**		**Multivariate analysis**	
	**HR (95% CI)**	***p*-value**	**HR (95% CI)**	***p*-value**
Gender (Male vs. Female)	1.16 (0.65–2.09)	0.617		
Age (≥64 vs. <64), year	1.35 (0.77–2.36)	0.290		
Current or former smoker	1.78 (1.03–3.10)	0.039	1.33 (0.63–2.82)	0.461
Pulmonary comorbidity	3.56 (1.92–6.59)	<0.001	1.13 (0.38–3.33)	0.825
FEV1/FVC (<70 vs. ≥70), %	5.33 (2.90–9.81)	<0.001	6.02 (2.13–17.00)	0.001
Cardiac comorbidity	3.21 (1.71–6.05)	<0.001	1.18 (0.45–3.10)	0.730
LVEF (<50 vs. ≥50), %	3.42 (1.85–6.32)	<0.001	3.38 (1.43–7.96)	0.005
LV diastolic dysfunction	2.35 (1.25–4.43)	0.007	1.70 (0.72–4.00)	0.224
ECOG performance status (≥1 vs. 0)	6.55 (2.97–14.42)	<0.001	4.38 (1.76–10.89)	0.001
Tumor location (Upper lobe vs. Lower/middle lobe)	0.72 (0.41–1.24)	0.235		
c-T stage(≥T3 vs. <T3)	1.40 (0.70–2.79)	0.336		
c-N stage (≥N2 vs. <N2)	1.33 (0.77–2.30)	0.313		
TNM stage (≥IIIA vs. <IIIA)	1.14 (0.59–2.21)	0.702		
Concurrent or sequential chemotherapy	2.66 (1.47–4.81)	0.001	3.08 (1.40–6.78)	0.005
Radiotherapy modality (3D-CRT vs. IMRT)	1.27 (0.73–2.22)	0.392		
Prescribed radiation dose (≥64 vs. <64), Gy	1.53 (0.88–2.66)	0.128		
Mean lung dose (≥16.5 vs. <16.5), Gy	3.39 (1.86–6.17)	<0.001	5.08 (2.39–10.82)	<0.001
Mean heart dose (≥12 vs. <12), Gy	1.34 (0.77–2.33)	0.297		

*FEV1/FVC, forced expiratory volume in the first second/forced vital capacity; LVEF, left ventricular ejection fraction; LV, left ventricular; ECOG, Eastern Cooperative Oncology Group; 3D-CRT, three-dimensional conformal radiation therapy; IMRT, intensity-modulated radiation therapy; HR, hazard ratio; CI, confidence interval*.

**Table 3:** Comparison of the risk of radiation pneumonitis between LVEF ≥50% and <50%.

	**Training set**		**Validation set**		**Training set**		**Validation set**	
					**Baseline dyspnea = 0**			
	**Grade 0–1 RP**	**Grade ≥ 2 RP**	**Grade 0–1 RP**	**Grade ≥ 2 RP**	**Grade 0–1 RP**	**Grade ≥ 2 RP**	**Grade 0–1 RP**	**Grade ≥ 2 RP**
LEVF ≥ 50%	159	42	76	23	83	13	37	10
LEVF < 50%	31	28	11	10	7	5	4	3
Odds ratio (95% CI)	3.42 (1.85–6.32)		3.00 (1.13–7.97)		4.56 (1.26–16.53)		2.78 (0.53–14.48)	
*p-*value	<0.001		0.023		0.014		0.213	

*LVEF, left ventricular ejection fraction; CI, confidence interval; RP, radiation pneumonitis*.

**Figure 1:**
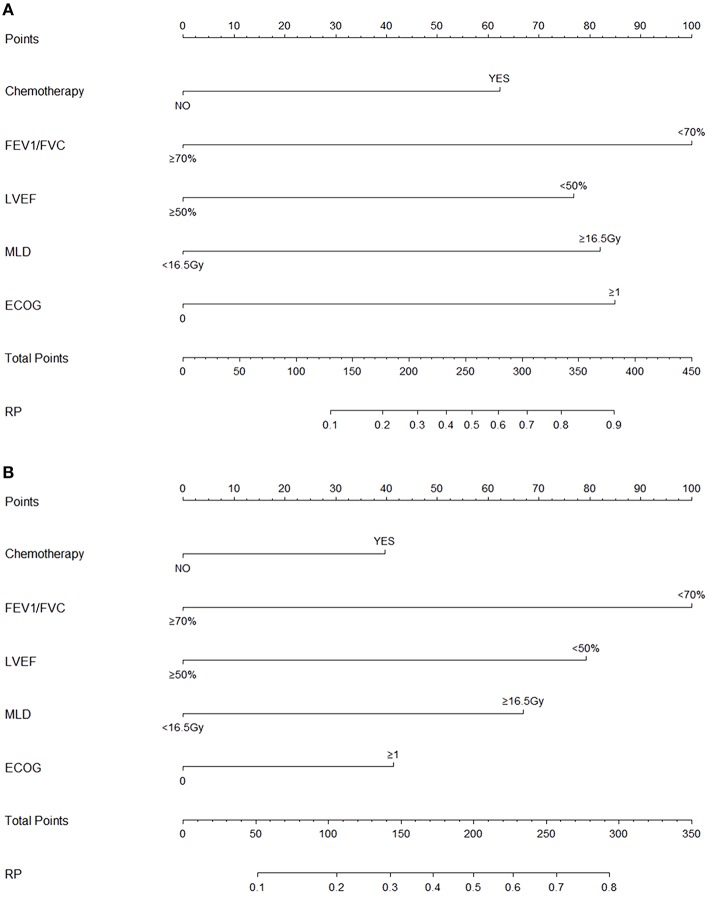
Nomograms for the model predicting grade ≥2 radiation pneumonitis on 10-fold cross-validation **(A)** and the external validation set **(B)**.

**Figure 2:**
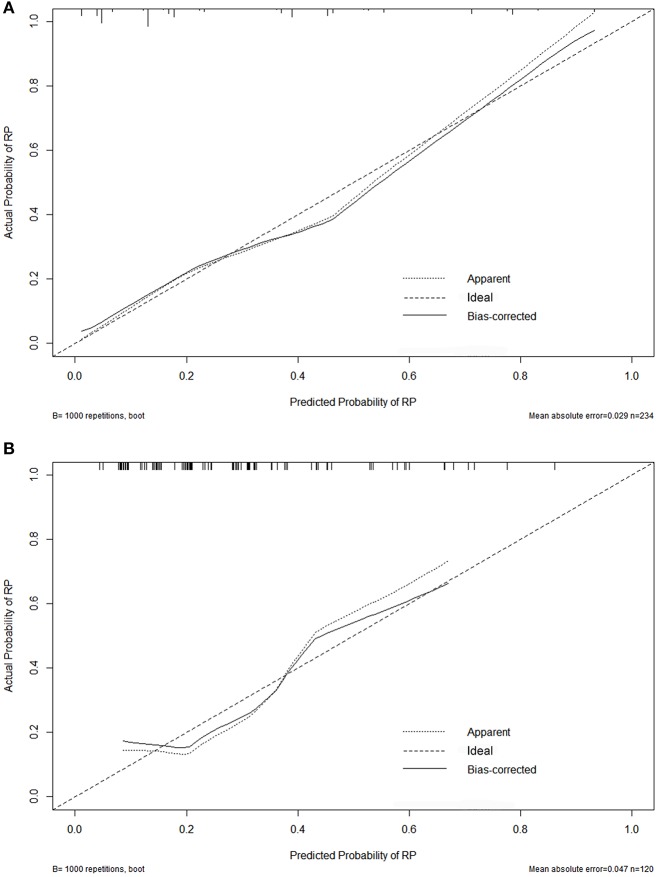
Calibration curves for the nomogram on 10-fold cross-validation **(A)** and the external validation set **(B)**.

**Figure 3:**
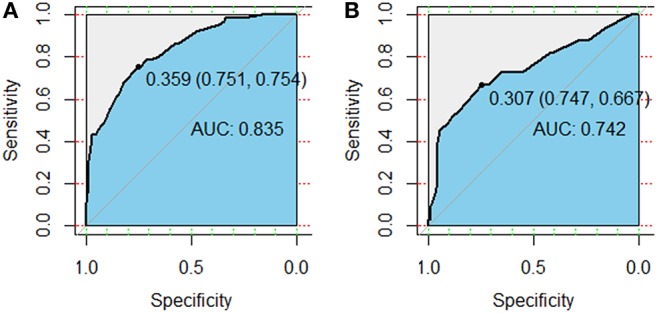
Receiver operator characteristic (ROC) curves for the model predicting grade ≥2 radiation pneumonitis on 10-fold cross-validation **(A)** and the external validation set **(B)**.

Variables including FEV1/FVC, LVEF, ECOG performance status, concurrent or sequential chemotherapy, and MLD were put into a model to predict grade ≥2 RP on 10-fold cross validation and the external validation set. The model demonstrated good discrimination (AUC 0.835, 95% CI, 0.778–0.891; *p* < 0.001) and the Hosmer-Lemeshow test confirmed good calibration (*p* = 0.656) on 10-fold cross validation. On the external validation set, the model remained strong predictive performance, maintaining an AUC of 0.742 (95% CI, 0.633–0.851; *p* < 0.001) and a Hosmer-Lemeshow test *p*-value of 0.534. The nomograms, calibration curves, and receiver operator characteristic curves for the model predicting grade ≥2 RP on 10-fold cross validation and the external validation set are shown in Figures 1–3, respectively.

In subgroup analysis, for patients with baseline dyspnea score of 0 (*n* = 108), reduced LVEF (HR, 4.67; 95% CI, 1.01–21.48; *p* = 0.048) was still significantly associated with an increased hazard of grade ≥2 RP (Table 4). However, these findings were not confirmed on the validation set, with a corresponding *p*-value of 0.213 (Table 3). Furthermore, compared with LVEF ≥50%, LVEF < 40% (HR, 2.37; 95% CI, 1.38–9.41; *p* = 0.009) was significantly associated with grade ≥2 RP, but LVEF in the range of 40–49% (HR, 2.37; 95% CI, 0.58–9.61; *p* = 0.228) did not significantly increase the risk of grade ≥2 RP (data not shown). Similar findings were noted on the external validation set: LVEF <40% (HR, 5.46; 95% CI, 1.14–26.12; *p* = 0.034) could increase the risk of grade ≥2 RP but LVEF of 40–49% (HR, 2.39; 95% CI, 0.36–15.75; *p* = 0.365) couldn't (data not shown). For grade ≥2 diastolic dysfunction, it was still not a significant factor (HR, 2.81; 95% CI, 0.81–9.78; *p* = 0.104) to predict the occurrence of grade ≥2 RP (data not shown).

**Table 4:** Multivariate logistic regressions analysis of grade ≥2 radiation pneumonitis for patients with baseline dyspnea = 0.

**Characteristic**	**Multivariate analysis**	
	**HR (95% CI)**	***p*-value**
Current or former smoker	1.52 (0.42–5.52)	0.523
Pulmonary comorbidity	0.35 (0.03–3.70)	0.380
FEV1/FVC (<70 vs. ≥70), %	6.77 (1.02–44.95)	0.048
Cardiac comorbidity	1.42 (0.20–10.00)	0.723
LVEF (<50 vs. ≥50), %	4.67 (1.01–21.48)	0.048
LV diastolic dysfunction	0.90 (0.13–6.27)	0.913
ECOG performance status (≥1 vs. 0)	3.03 (0.74–12.43)	0.124
Concurrent or sequential chemotherapy	2.38 (0.61–9.32)	0.215
Mean lung dose (≥16.5 vs. <16.5), Gy	6.77 (1.02–44.95)	0.048

*FEV1/FVC, forced expiratory volume in the first second/forced vital capacity; LVEF, left ventricular ejection fraction; LV, left ventricular; ECOG, Eastern Cooperative Oncology Group; HR, hazard ratio; CI, confidence interval*.

## Discussion

In the present study, we assessed the relationship between LV systolic and diastolic function at baseline and grade ≥2 RP, demonstrating that LVEF <50% was a possible independent risk factor of grade ≥2 RP for locally advanced lung cancer patients treated with definitive radiotherapy. This may be a surprising finding, for there have been no reports to assess the relationship between LV systolic and diastolic dysfunction and RP. These data may inform radiation oncologists that for patients with LV systolic dysfunction, especially for those with LVEF <40%, individualized radiotherapy and frequent monitoring of pulmonary status after radiotherapy should be considered. Given that previous studies had demonstrated that heart irradiation was an independent risk factor for RP (12), we used a chi-square test to assess whether MHD might be a confounding factor, and therefore patients with higher MHD were actually those who developed RP; a *p*-value of 0.297 suggested that there had been no relationship between MHD and grade ≥2 RP.

LVEF, calculated by end-diastolic volume-end-systolic volume/end-diastolic volume, is the most validated and commonly used echocardiographic measure of systolic function (24). A cut-off of 50% is usually defined as a normal limit for LVEF (25). LVEF is a reliable method to measure both LV contractile function and structure and to identify heart failure therapeutic phenotypes. It is also the best current method of assessing pathologic remodeling in heart failure (26). Ventricular systolic dysfunction begins with a reduction in systolic pump function following a loss of muscle cells, a decrease in myocardial contractility, and/or structural changes of the myocardium with an increase in interstitial fibrosis (27). The reduced systolic pump function induces several compensatory mechanisms including the Frank-Starling principle, neurohormonal activation of the sympathetic nervous system, and the renin-angiotensin–aldosterone system increases the strength of subsequent ventricular contraction and the stroke volume. However, the compensatory mechanisms can also lead to an enlargement of ventricle and an increase in end-diastolic pressure (27, 28). The adverse effects of these compensatory mechanisms are reduced by subsequent myocardial hypertrophy, activation of the baroreceptor reflex, and release of atrial natriuretic peptide (27). However, with the reduction of cardiovascular reserve, ventricular contraction, and stroke volume, LVEF is reduced and end diastolic volume is increased, which ultimately could lead to dyspnea and other congestive symptoms (28). The detailed mechanism regarding reduced LVEF on the development of RP is not clear, possibly related to the detrimental effect of LV systolic dysfunction on lung tissue, and it should be elucidated in the future. However, using new diagnostic techniques of myocardial deformation, several studies have shown subtle changes in systolic function that couldn't be detected by ejection fraction (29–31). Additionally, some studies demonstrated that there was a slow but progressive decline in LVEF in patients with diastolic heart failure, and these patients would eventually be diagnosed with heart failure with reduced LVEF (32, 33). Therefore, there may be inaccuracies in evaluating LV systolic function by LVEF, and thus more sensitive measure for evaluating LV systolic function, such as global longitudinal strain (34), may be needed in the future study.

According to the 2016 European Society of Cardiology guidelines (25), heart failure with LVEF of 40–49% is named with heart failure with mid-range ejection fraction (HFmrEF). The underlying pathophysiology of HFmrEF is not completely clear (35). The 2016 European Society of Cardiology guidelines suggest that patients with HFmrEF probably have mild systolic dysfunction as well as diastolic dysfunction (25). A recent study divided HFmrEF patients into three subgroups: HFmrEF improved (prior LVEF < 40%), HFmrEF deteriorated (prior LVEF > 50%), and HFmrEF unchanged (prior LVEF 40–50%) and found heterogeneity in epidemiology, pathophysiology, and clinical outcomes between these subgroups (36). In our study, we found that compared with LVEF ≥ 50%, LVEF in the range of 40–49% couldn't significantly increase the risk of grade ≥2 RP, possibly due to the heterogeneity between patients with mid-range LVEF. Additionally, this study demonstrated that for patients with baseline dyspnea score of 0 (*n* = 108), reduced LVEF was still significantly associated with an increased hazard of grade ≥2 RP. However, these findings were not confirmed on the validation set, suggesting that the relationship between reduced LVEF and grade ≥2 RP for asymptomatic patients is not clear and corresponding further studies with large population size are needed to clearly determine this relationship.

Seeking a suitable diagnostic criteria for LV diastolic dysfunction doesn't seem to be an easy task. The diagnostic criteria of LV diastolic dysfunction by echocardiography is changing. The 2009 consensus guidelines from the American Society of Echocardiography and the European Association of Echocardiography on evaluating diastolic dysfunction use three variables: septal and lateral e′ velocity and LAVI to determine whether patients have diastolic dysfunction (37). According to the 2009 consensus guidelines, there should be eight (2 × 2 × 2) possible combined results from the three variables, but the guidelines include only three combined results, ignoring the other five, which could lead to many indeterminate assessments for diastolic function. The 2009 consensus guidelines provide a low sensitivity of 47% for identifying diastolic dysfunction in heart failure with preserved ejection fraction patients (38). The updated 2016 joint guideline from the American Society of Echocardiography and the European Association of Cardiovascular Imaging selects four variables: annular e′ velocity, average E/e′ ratio, LAVI, and peak tricuspid regurgitation velocity to determine the presence of diastolic dysfunction (39). However, using this updated joint guideline to evaluate diastolic function could also create a situation that diastolic dysfunction is underdiagnosed. For example, if 50% of the above four variables are positive, then diastolic dysfunction would be considered indeterminate under this classification scheme. Additionally, increased peak TR velocity is not merely determined by an elevation in LV filling pressure, and can be caused by a pre-capillary component of pulmonary hypertension. Increased peak TR velocity caused by an elevation in LV filling pressure is seen in advanced stages of diastolic dysfunction. Therefore, patients with early diastolic dysfunction may be underdiagnosed (18). Mitter et al. (18) recently put forward a new algorithm for diagnosing diastolic dysfunction with Doppler echocardiography. Although this algorithm lacks validation in clinical practice, it selects commonly used variables to assess diastolic function from the commonly used 2009 consensus guidelines. Notably, this algorithm deems that a LAVI of >28 ml/m^2^ can suggest early diastolic dysfunction and that criteria for an abnormal lateral e′ used to evaluate diastolic function should be population-based and age-related. Furthermore, this algorithm helps echocardiogram readers to distinguish pitfalls quickly, limiting adequate evaluation of diastolic function (18). Therefore, our study adopted this algorithm to assess LV diastolic function.

Cardiac comorbidity was also selected for the univariate analysis in our study. But unlike with Nalbantov et al. (14), we found that cardiac comorbidity was significantly associated with grade ≥2 RP in univariate analysis but didn't remain significant difference in multivariate analysis, possibly due to a lack of timeliness for the history of cardiac comorbidity: pre-existing cardiac disease couldn't represent the cardiac status on admission, and patients may have underlying cardiac disease on admission that was not previously discovered.

Previous studies have demonstrated that among the first-line chemotherapy regimens for lung cancer, paclitaxel, gemcitabine, and vinorelbine can lead to pulmonary toxicity (40, 41), and there was no sufficient evidence to confirm that other drugs can cause pulmonary toxicity. Therefore, we divided patients who used paclitaxel, gemcitabine, and vinblastine into one group and divided other patients into another group, investigating whether chemotherapy drugs that can cause pulmonary toxicity has an effect on the occurrence of RP. The results revealed that *p*-values for the training and validation sets were 0.364 (HR, 1.34; 95% CI, 0.73–2.41) and 0.286 (HR, 1.58; 95% CI, 0.86–2.79), respectively (data not shown), indicating that there was no association between chemotherapy drugs that can cause pulmonary toxicity and RP.

There were several other limitations in this study. First, the retrospective nature may lead to a bias during patients' selection and inaccuracies of data. Second, the algorithm we used to evaluate diastolic dysfunction has not been validated clinically, and it may yield a low specificity for identifying diastolic dysfunction owing to the low normal limit of LAVI (28 ml/m^2^). Last, other biomarkers commonly used to evaluate cardiac function, such as brain natriuretic peptide and N-terminal pro-brain natriuretic peptide (42), were not factored into this study. Newer echocardiographic parameters for assessing diastolic function, specifically left atrial strain (43, 44), have been reported in recent years. Therefore, further prospective studies with indicators more accurately reflecting LV systolic and diastolic functions are needed to confirm and broadly interpret the present findings.

In conclusion, LV systolic dysfunction is a possible independent risk factor of grade ≥2 RP for locally-advanced lung cancer patients receiving definitive radiotherapy. Compared with LVEF ≥50%, LVEF <40% is significantly associated with grade ≥2 RP, but LVEF in the range of 40 to −49% couldn't increase the risk of grade ≥2 RP. For asymptomatic patients, it remains unclear whether systolic dysfunction has an effect on the development of grade ≥2 RP and should be elucidated in the future.

## Data Availability Statement

The datasets generated for this study are available on request to the corresponding author.

## Ethics Statement

The studies involving human participants were reviewed and approved by The Ethics Committee of Qilu Hospital of Shandong University, The Ethics Committee of Shandong Provincial Hospital. The patients/participants provided their written informed consent to participate in this study. Written informed consent was obtained from the individual(s) for the publication of any potentially identifiable images or data included in this article.

## Author Contributions

XM and JY contributed to the conception and design of the study. GC and CL organized the database. GC and SL performed the statistical analysis. GC and XM wrote the first draft of the manuscript. All authors contributed to manuscript revision, read and approved the submitted version.

### Conflict of Interest

The authors declare that the research was conducted in the absence of any commercial or financial relationships that could be construed as a potential conflict of interest.

## Abbreviations

NSCLC: non-small cell lung cancer
RP: radiation pneumonitis
LV: left ventricular
LVEF: left ventricular ejection fraction
ECOG: Eastern Cooperative Oncology Group
MLD: mean lung dose
MHD: mean heart dose
FEV1/FVC: forced expiratory volume in the first second/forced vital capacity
LAVI: left atrial volume index
e′: lateral early diastolic mitral annular velocity
E/A: the ratio of early diastolic transmitral flow velocity to late diastolic transmitral flow velocity
AUC: area under the receiver operating characteristic curve
HR: hazard ratio
CI: confidence interval
HFmrEF: heart failure with mid-range ejection fraction.
